# Expression of Concern: CircRNA circPDSS1 promotes bladder cancer by downregulating miR-16

**DOI:** 10.1042/BSR-20191961_EOC

**Published:** 2021-04-06

**Keywords:** circPDSS1, miR-16, urothelial bladder cancer

The Editorial Office has been made aware of potential issues surrounding the scientific validity of this paper, hence has issued an expression of concern to notify readers whilst the Editorial Office investigates.

